# Design and Volatile Compound Profiling of Starter Cultures for Yogurt Preparation

**DOI:** 10.3390/foods12020379

**Published:** 2023-01-13

**Authors:** Albert Krastanov, Marin Georgiev, Aleksandar Slavchev, Denica Blazheva, Bogdan Goranov, Salam A. Ibrahim

**Affiliations:** 1 Department of Biotechnology, University of Food Technologies, 4002 Plovdiv, Bulgaria; 2 Department of Microbiology, University of Food Technologies, 4002 Plovdiv, Bulgaria; 3 Food Microbiology and Biotechnology Laboratory, Food and Nutritional Science Program, North Carolina A&T State University, Greensboro, NC 27411-1064, USA

**Keywords:** Bulgarian yogurt, starter cultures, biocompatibility, volatile compound profile

## Abstract

Stable symbiotic starter cultures were created using selected strains of *Lactobacillus delbrueckii* subsp. *bulgaricus* and *Streptococcus salivarius* subsp. *thermophilus* with antimicrobial activity against pathogens and necessary antibiotic sensitivity, growth kinetic parameters, and metabolic profiles. The volatile compound profiles of the obtained starter cultures were determined and their specificity was proven depending on the ratio of monocultures in each combination. The influence of the freeze-drying process on the starter cultures in relation to the production of aromatic components was investigated and it was demonstrated that this process had a significant effect on the content of the aroma-forming substances in the fermented milk. However, the influence of the pre-cooling process and crude fat content from 1.5 to 3.0% did not notably affect the levels of volatile compounds synthesized by the selected starter cultures. Comprehensive data for all volatile aromatic metabolites in the fermented milk were also obtained. These designed symbiotic starter cultures can be used to produce traditional Bulgarian yogurt with increased functional and probiotic properties.

## 1. Introduction

Fermentation was one of the first methods employed by humans to extend the shelf life of food. Yogurt, under various names and in different forms, is a staple in the diet of many cultures. Generally, it is made from milk fermented with lactic acid bacteria [1].

At the beginning of the 20th century, Nobel Prize winner Ilya Metchnikov attributed the longevity of Bulgarians to the traditionally prepared yogurt in their diets [2]. This sparked a worldwide interest in yogurt and a significant increase in its consumption. In order for a dairy product to be labeled as Bulgarian yogurt, it should comply with a number of requirements, including the bacterial species in the starter culture, the concentration of viable cells, and the ratio between the species. The starter culture should include only *Lactobacillus delbrueckii* subsp. *bulgaricus* and *Streptococcus salivarius* subsp. *thermophilus* in a certain ratio and (at least) 10^7^ cfu/g total viable cell count at the end of the product shelf life [3].

Numerous health benefits, such as improvement in gastrointestinal issues like constipation, diarrhea, and lactose intolerance symptoms [4,5,6]; anticancer properties; reduction of cholesterol; immune system stimulation [7,8]; beneficial effects on type 2 diabetes and obesity patients [9]; mood improvement [10]; resistance to influenza virus infection in mice [11]; etc. are attributed to the consumption of yogurt.

However, these are not the only reasons for the popularity of yogurt around the world. Yogurt also has sensory characteristics such as a desirable flavor that make it attractive to consumers. A major factor in the development of the desirable taste and aroma of yogurt is the starter culture. Through a series of biochemical transformations of the carbohydrates, fats, and proteins in milk during the fermentation process, the lactic acid bacteria form multiple flavor compounds which contribute to the formation of a unique organoleptic profile for each starter culture.

The content of volatile compounds in yogurt can vary significantly. For example, more than 90 different substances have been identified in different products [12,13,14], such as organic acids, ketones, aldehydes, alcohols, esters, and hydrocarbons [10,15]. A suitable method for the quantification of flavor is analysis based on solid-phase microextration (SPME) combined with gas chromatography-mass spectrometry (GC-MS). This method has been successfully used for the flavor profiling of dairy products [16,17,18,19] and other fermented foods [20,21]. It provides data that can be used for the precise selection of starter cultures that produce yogurt with specific organoleptic properties (e.g., according to consumer preferences) and for quality and authenticity control.

Despite the enormous importance of lactic acid bacteria, very little is known about the mechanisms of their contribution to flavor formation in food products subjected to lactic acid fermentation. Advances in the development of instrumental methods now allow researchers to avoid some difficulties in the extraction of the aroma-forming substances from the otherwise complex matrix of fermented dairy products. Using synthetic fiber solid phase extraction (SPME) and gas chromatography combined with mass spectrometry (GC-MS), a large number of volatile compounds can be investigated, isolated, and identified [16,17,18,19,20,21]. These techniques open up the potential for the more comprehensive and advanced investigation of the importance of specific strains in obtaining the desired sensory qualities of fermented milk products. There are also insufficient data on the importance of certain technological parameters and strain specificity for aroma formation in yogurt. Such additional knowledge would help in the selection of production strains and the provision of the best flavor-producing symbiotic cultures.

At a more advanced level, these analyses could facilitate the development of rapid methodologies for the quality and authenticity control of dairy products based on the aroma-metabolite profile of the starter cultures in the final product.

In the current study, we present a method for the design and characterization of symbiotic starter cultures of new strains of *Lactobacillus delbrueckii* subsp. *bulgaricus* and *Streptococcus salivarius* subsp. *thermophilus* for fermented milk products. An analysis of the metabolic profile of symbiotic starter cultures and a comparison of the metabolic profiles of lyophilized starter cultures for direct application with that of a production (liquid) culture are also provided. In addition, we investigate the influence of the pre-cooling stage and crude fat content on the aromatic-metabolic profile of the studied symbiotic cultures.

## 2. Materials and Methods

### 2.1. Microorganisms

Nine *Lactobacillus delbrueckii* subsp. *bulgaricus* strains (*Lactobacillus bulgaricus* MG1, *Lactobacillus bulgaricus* MG2, *Lactobacillus bulgaricus* MG3, *Lactobacillus bulgaricus* MG4, *Lactobacillus bulgaricus* MG5, *Lactobacillus bulgaricus* MG6, *Lactobacillus bulgaricus* MG7, *Lactobacillus bulgaricus* MG8, and *Lactobacillus bulgaricus* MG9) and eight *Streptococcus salivarius* subsp. *thermophilus* strains (*Streptococcus thermophilus* T1, *Streptococcus thermophilus* T2, *Streptococcus thermophilus* T3, *Streptococcus thermophilus* T4, *Streptococcus thermophilus* T5, *Streptococcus thermophilus* T6, *Streptococcus thermophilus* T7, and *Streptococcus thermophilus* T8) were used in the experiments. They were isolated from homemade dairy products from ecologically clean areas in Bulgaria—Stara Planina, Rhodopes, Rila, Devin, Predela, and Shiroka Polyana. The strains were maintained in 10% sterile reconstituted powder milk after cultivation under static conditions for 12 h at a temperature of 44 ± 1 °C for the *L. bulgaricus* strains and 42 ± 1 °C for the *S. thermophilus* strains.

### 2.2. Determination of Biocompatibility between the Selected Strains

In order to determine biocompatibility, a method adapted from Glushanova (2005) [22] was used; 24 h cultures cultivated in LAPTg10 broth (composition, g/dm^3^: peptone—15; yeast extract—10; tryptone—10; glucose—10, Tween 80—1 cm^3^/dm^3^; pH 6.6 ± 6.8) were used and 5 μL *L. bulgaricus* cell suspension of each culture was micropipetted onto LAPTg10 agar plates (composition, g/dm^3^: peptone—15; yeast extract—10; tryptone—10; glucose—10, Tween 80—1 cm^3^/dm^3^; pH 6.6 ± 6.8, agar-agar—15). After the liquid diffused into the agar (20–30 min) at a distance of 1–2 mm from the end of the first drop, 5 μL of the second tested strain was placed and time was allowed for the liquid to diffuse into the agar. The inoculated Petri dishes were incubated at 42 °C and checked for biocompatibility of the strains at the 24th and 48th hours. If the growth of one strain was inhibited by the other, their relationship was antagonistic, and the cultures were strongly bioincompatible. Biocompatibility of the tested strains was determined by whether complete fusion of the growth zones was observed. Each *Lactobacillus* strain was tested against each *Streptococcus* strain.

### 2.3. Preparation of Symbiotic Starter Cultures from Lactobacillus delbrueckii *subsp.* bulgaricus and Streptococcus thermophilus for Traditional Bulgarian Yogurt

The biocompatible combinations were investigated to confirm the symbiotic relationships between lactobacilli and streptococci. The test combinations were used daily for consecutive inoculations of 10% reconstituted powder milk for 4 days. After coagulation on the 4th day, the fermented milk was stored for 10 days at 4 °C and used for consecutive inoculations daily for another 4 days. The content of viable *L. bulgaricus* and *S. thermophilus* cells was determined by cultivation on MRS and M17 agar after appropriate dilutions in a 0.5% saline solution and the ratio of streptococci to lactobacilli was calculated. The coagulation time of the obtained yogurts was determined visually and the titratable acidity analysis was conducted according to the Thorner method [23].

### 2.4. HS-SPME-GC-MS Analysis

Headspace solid-phase microextraction–gas chromatography-mass spectrometry (HS-SPME-GC-MS) was used to analyze the volatile compounds produced by each of the tested combinations of lactobacilli and streptococci. Toluene (Sigma-Aldrich Corp., Burlington, MA, USA) was used as an internal standard. In 20-mL glass vials, 5 mL of the sample was mixed with the internal standard to a final concentration in each sample of 10 µg/L. The samples were heated at 50 °C and, subsequently, an SPME fiber (50/30 um DVB/Carboxen/PDMS; Supelco Inc., Bellefonte, PA, USA) was exposed in the headspace for 50 min. The fiber was then immediately inserted into the injection port of a Trace 1300 GC (Thermo Fisher Scientific) for 5 min at 270 °C to desorb volatile compounds into the GC. The optimum extraction conditions were selected based on preliminary experiments on SPME extraction of samples of starter culture KM1 (*L. bulgaricus* MG3 and *S. thermophilus* T1) at different extraction temperatures (40 °C, 50 °C, 60 °C, and 70 °C) and time intervals (30, 40, 50, and 60 min).

The volatile compounds synthesized by each starter culture were identified using a Trace 1300 GC equipped with an ISQ QD, Single Quadrupole Mass Spectrometer (both Thermo Fisher Scientific Inc., Waltham, MA USA) equipped with a TR5MS column (length, 30 m; i.d., 0.25 mm; film thickness, 0.25 µm; Thermo Fisher Scientific). Helium was used as the carrier gas at 1 mL/min. The GC temperature was initially maintained at 35 °C for 5 min and then increased to 140 °C at a rate of 4 °C/min for 5 min, heated to 270 °C at a rate of 10 °C/min, and, finally, held at 270 °C for 5 min. The mass detection was made according to the manufacturer’s recommendations in full scan mode. The ion source and transfer line temperatures were 220 °C and 260 °C, respectively. The mass spectra from each sample were recorded using a scan range of 40–400 m/z. Each sample measurement was carried out in triplicate.

### 2.5. Lyophilization of the Resulting Symbiotic Starter Cultures

Lyophilization was performed using a Lyovac GT2 apparatus (Laybold GmbH, Cologne, Germany). The starter cultures were pre-frozen to −40 °C. Lyophilization occurred over 48 h at a working pressure of −25 Pa. The process ended at a post-drying temperature of 35 °C. A 15% sucrose solution was used as a cryoprotectant.

### 2.6. Determination of the Concentration of Viable Cells

The concentration of viable cells of the studied strains of *L. bulgaricus* and *S. thermophilus* was determined by the tenfold dilution method according to ISO 6091:2010 [24]. In brief, after appropriate serial tenfold dilutions in 0.5% saline, the samples were inoculated in MRS (for *L. bulgaricus*) and M17 (for *S. thermophilus*) agar.

### 2.7. Determination of Titratable Acidity

The titratable acidity was determined by the Thorner method according to the requirements of BDS 1111:1980 [23] by titration with NaOH (0.1 N) 0.1% using phenolphthalein.

### 2.8. Statistical Analysis

All the experiments were conducted in triplicate and the values were expressed as means. Statistical significance was detected by analysis of variance (ANOVA, Tukey’s test; the value of *p* < 0.05 indicated statistical difference).

## 3. Results and Discussion

### 3.1. Preparation and Characterization of Symbiotic Starter Cultures from Lactobacillus delbrueckii *subsp.* bulgaricus and Streptococcus salivarus *subsp.* thermophilus for Traditional Bulgarian Yogurt

The *Lactobacillus* and *Streptococcus* strains were subjected to a biocompatibility test according to an adapted version of the Glushanova method [22] (Table 1).

As a result of these studies, the following combinations were selected: KM1 (*L. bulgaricus* MG3 and *S. thermophilus* T1), KM2 (*L. bulgaricus* MG7 and *S. thermophilus* T1), KM3 (*L. bulgaricus* MG9 and *S. thermophilus* T1), KM4 (*L. bulgaricus* MG3 and *S. thermophilus* T2), KM5 (*L. bulgaricus* MG7 and *S. thermophilus* T6), and KM6 (*L. bulgaricus* MG9 and *S. thermophilus* T6). In these tests, complete biocompatibility between the studied lactobacilli and streptococci was observed in combinations KM1, KM2, and KM4. The relationship between the *L. bulgaricus* and *S. thermophilus* strains in combinations KM3, KM5, and KM6 was at the biocompatibility limit. The resulting combinations were investigated to confirm the symbiotic relationships between lactobacilli and streptococci.

Stable symbiotic starter cultures were formed in combinations KM1, KM2, and KM4, which confirmed the biocompatibility test results. The yogurts obtained with combination KM1 contained a high concentration of active cells (10^9^ cfu/g lactobacilli and 10^10^ cfu/g streptococci) and a stable symbiosis occurred from the first days of the experiment. The ratio between rods and cocci in this combination was close to 1:3 in favor of *S. thermophilus*. The symbiotic relationship between the strains in the starter culture was evidenced by the rapid coagulation time, which ranged from 3 h to 3 h 15 min. The titratable acidity of the obtained yogurt was between 61 to 70 °T at the time of coagulation and increased from 70 to 84 °T after 24 h (Tables S1 and S2).

The concentration of active cells of *L. bulgaricus* and *S. thermophilus* in the yogurt obtained with the KM2 combination was almost an order of magnitude lower than the one fermented with the KM1 combination. In this combination, the occurrence of stable symbiosis was also observed from the beginning of the experiment, but in contrast to the KM1 combination, in KM2 the ratio of rods: cocci was approximately 1:9 in favor of streptococci. The coagulation time of yogurts fermented with the KM2 combination was in the range of 2 h 40 min to 2 h 45 min and after 10 days of storage of the starter culture, the coagulation time was 3 h. The shorter coagulation time in this starter culture can be explained by the higher ratio in favor of streptococci, which depleted the dissolved oxygen faster, lowered the pH, and created appropriate conditions for the development of *L. bulgaricus*. Additionally, *L. bulgaricus* MG7 was the most active acid-producing of all studied strains. The titratable acidity of the yogurt obtained with the KM2 combination ranged from 66 to 68 °T at the time of coagulation and from 86 to 88 °T after 24 h. The results for this combination also coincided with the biocompatibility test (Tables S3 and S4).

The tests showed that the strains in combination KM4 were biocompatible and the combination was also subjected to consecutive inoculations to confirm symbiosis. A stable symbiosis was established in this combination from the beginning of the experiment. The ratio of rods to cocci was close to 1:5 in favor of the streptococci. This combination achieved a high content of lactobacilli in the range of 10^10^ cfu/g and streptococci—10^11^ cfu/g. The milk coagulated after 3 h 10 min on the 1st and 14th days of the experiment and after 3 h on the other days. The titratable acidity of the yogurt varied from 61 to 66 °T at the time of coagulation and from 78 to 80 °T after 24 h (Tables S7 and S8).

During the first stage of consecutive inoculations, the strains in starter culture KM3 were on the border between compatibility and incompatibility. The ratio of lactobacilli to streptococci varied from 1:250 in favor of cocci on the 1st day to nearly 1:1 (1:1.4) on the 4th day. The number of active cells of both lactobacilli and streptococci varied from 10^7^ to 10^9^ cfu/g for *L. bulgaricus* and from 10^9^ to 10^10^ cfu/g for *S. thermophilus*. The coagulation time of milk with this combination during the first 4 days of daily inoculations was 3 h 25 min and the titratable acidity varied from 62 to 65 °T at the time of coagulation and from 68 to 74 °T after 24 h. However, after 10 days of refrigerated storage and during the second stage of daily consecutive inoculations, a symbiotic relationship between lactobacilli and streptococci was established. In the second stage of inoculations, the ratio of rods to streptococci was already stable—close to 1:9 (1:8.6 and 1:8.7). The same trend was observed in the number of active cells of lactic acid bacteria. During the second stage of the experiment, the concentration of active cells was of the order of 10^9^ cfu/g for *L. bulgaricus* and 10^10^ cfu/g for *S. thermophilus*. There was also a reduction in coagulation time to 3 h. The titratable acidity at 24 h varied in the range of 82 to 84 °T. When performing the biocompatibility test for the strains from this combination, the result was borderline. However, these strains, although at a later stage, managed to form a symbiotic relationship (Tables S5 and S6).

The strains in combinations KM5 and KM6 failed to form stable symbiotic relationships (Tables S9–S12). In combination KM5, there was even strong antagonism between *L. bulgaricus* and *S. thermophilus*, which was characterized by complete suppression of the growth of *L. bulgaricus* and its complete expulsion by *S. thermophilus* (Tables S9 and S10).

### 3.2. Determination of the Metabolic Profiles of the Obtained Symbiotic Starter Cultures

The aromatic metabolic profiles of the four symbiotic starter cultures were determined. They were cultivated in reconstituted milk powder. During the chromatographic analysis, 47 aromatic components were identified in the different starter cultures. These metabolites were grouped into classes depending on their chemical structure and are presented in Table 2.

- Aldehyde components

Of the aldehyde components involved in flavor, eight components were identified in yogurts obtained with starter cultures KM1, KM2, KM3, and KM4. Acetaldehyde, furaldehyde, 3-hydroxybutanal, benzaldehyde, benzacetaldehyde, ethylbenzaldehyde, and 2-octanal were detected in all starter cultures. The presence of decanal was detected only in the KM4 starter culture due to the activity of *S. thermophilus* T2 in the composition of this starter culture, which has the ability to produce this metabolite [25].

Of the aldehyde components, benzaldehyde had the highest concentrations in all four starter cultures. Of the four tested starter cultures, higher concentrations of benzaldehyde were produced from the KM3 starter culture, followed by KM4. Lower and comparable concentrations of benzaldehyde were observed in yogurts obtained with starter cultures KM1 and KM2. The second aromatic component that was found in higher concentrations than the others was acetaldehyde. The highest concentration was found in starter culture KM1. Lower and closer concentrations of acetaldehyde were observed in starter cultures KM2 and KM3 and the lowest concentration of this metabolite was found in KM4.

It is noteworthy that in the yogurts obtained with starter culture KM1, the synthesis of furaldehyde was observed, which was not produced by the monocultures that comprised the symbiotic starter [25], and it was the highest concentration compared to the other starter cultures. The symbiosis between the strains in KM1 likely led to the production of this component.

- Ketone components

From the group of ketones, eight compounds were identified in yogurts obtained with the studied starter cultures. The metabolites with higher relative peak areas and respectively in higher concentrations were acetoin, 2-pentanone, 2,3-butanedione, and 2-nonanone. Of these, acetoin had the highest concentration, of which starter culture KM4 produced the most.

Very similar concentrations of 2,3-butanedione were observed for KM1, KM3, and KM4, where the relative areas of the peaks were commensurate. A lower concentration of this metabolite was observed in the KM2 starter culture.

In yogurts fermented with starter cultures KM1, KM3, and KM4, 2-nonanone was found in very similar concentrations. No 2-nonanone was detected in KM2, despite the fact that the monocultures of which KM2 was composed of produce it [25]. Most likely, this compound underwent chemical modification and changed its structure or entered into chemical interactions with other substances during the mixed cultivation of lactobacilli and streptococcal strains, which is why it was not detected on the chromatogram. A similar trend was observed for 3-methyl-2-butanone, which was absent in the chromatogram of the aromatic profile for starter culture KM1. The other aromatic metabolites of the ketone group, namely, 2-acylfuran, 2-heptanone, 2-undukanone, and 3-methyl-2-butanone, were in significantly lower concentrations.

- Organic acids

Acetic and hexanoic acids were dominant acidic compounds and respectively formulated the aromatic profile of yogurts obtained with the studied starter cultures to the largest extent. For the four starter cultures, very close values of the relative area of the peaks were observed, which indicates comparable concentrations of acetic acid in yogurts.

The data presented in Table 3 show that KM4 produced more heptanoic acid than the rest of the starter cultures, followed by KM3 and KM1. KM2 was characterized by a lower concentration of hexanoic acid compared to the other three starter cultures. In yogurt fermented with starter culture KM4, the highest content of formic acid was observed, while in the other three starter cultures, the amount of formic acid was significantly lower and similar between the individual cultures.

Pentanoic acid, benzoic acid, octanoic acid, 1,2-benzaldehydecarboxylic acid, and butyric acid, which was not found in monocultures of lactobacilli and streptococci [25], were also found in the studied starter cultures. The largest amount of butyric acid was produced by starter culture KM4, followed by starter culture KM2. An intermediate in terms of its ability to produce butyric acid was KM1. Starter culture KM3 produced butyric acid in a lower concentration compared to other starter cultures. Over the last decade, scientists have focused on studying the physiological and probiotic effects of butyric acid on the human body. Numerous studies by a number of authors have shown that butyric acid, along with its antimicrobial activity, serves as an energy source for colonocytes, restores the integrity of the epithelial cells of the intestinal mucosa, reduces inflammation of the intestinal tissue, and has immunomodulatory effects. In addition, butyric acid inhibits the growth of cancer cells and affects recovery from Crohn’s disease. Butyric acid, synthesized by probiotic bacteria, improves erythrocyte circulation and neutralizes carcinogens such as nitrosamines, which result from putrefactive processes of unwanted toxicogenic microflora in the gastrointestinal tract, especially in people on a high-protein diet. Due to these properties of butyric acid, the use of lactic acid bacteria that synthesize butyric acid is especially relevant, both as probiotics in medical practice and in the composition of starter cultures to obtain various fermented dairy products with increased functional properties [26,27,28]. In this regard, starter cultures KM4 and KM2 are particularly suitable for the production of functional dairy products as they synthesize the largest amounts of butyric acid compared to the other two starter cultures. Pentanoic acid, benzoic acid, octanoic acid, and 1,2-benzaldehydecarboxylic acid in different starter cultures were produced in different concentrations.

- Alcohol components

The main alcoholic components which formed the aromatic profile of fermented milk with the studied starter cultures were 2-furanmethanol, 2-octoxyethanol, and 3-methyl-2-butanol as they were in significantly higher concentrations than 2-undecanol. Of these components in all four starter cultures, 2-furanmethanol was dominant. A larger amount of 3-methyl-2-butanol was produced by starter culture KM3, followed by KM1 and KM4. The lowest concentration of this metabolite was found in KM2-fermented yogurt.

- Ester components

In the yogurts obtained with the studied symbiotic starter cultures, new ester components were detected, which were absent in the monocultures of lactobacilli and streptococci [25]. These were ethenyl propanoate, tridec-2-ynyl 3-methylbut-2-enoate, and 1-(2,4-dimethylphenyl)-ethanone. Of these three new ester compounds, ethenyl propanoate was found in the largest amount. Starter culture KM3 produced more of this metabolite than other cultures. Next in terms of the amount of compound produced were KM2 and KM4, which synthesized very similar concentrations of the metabolite. KM1 had a lower amount of this metabolite. Tridec-2-ynyl 3-methylbut-2-enoate was produced in larger amounts by KM3 and KM1. This metabolite was produced at lower concentrations by KM2 and KM4.

Starter cultures KM1, KM2, and KM4 produced similar concentrations of 1-(2,4-dimethylphenyl)-ethanone, and higher concentrations of this metabolite were found in milk fermented with KM3. 2-Methyl-2H-furan-5-one was detected only in yogurt obtained with starter culture KM2. The relative peak areas for 2-ethylhexyl benzoate were very close in starter cultures KM1, KM3, and KM4, which suggested close concentrations of the metabolite in milk obtained with these cultures. A lower concentration of the aromatic component was observed in KM3. Starter cultures KM1 and KM3 produced higher and similar concentrations of methyl 4-ethylbenzoate. Lower and also similar concentrations of this metabolite were observed for the other two starter cultures.

- Aromatic hydrocarbons

In the yogurts fermented with the studied starter cultures, 13 components from the aromatic hydrocarbons group were identified. Of these, newly detected compounds were identified that were not observed in the monocultures. These were 2,4,4-trimethylpent-2-ene, nonadecane, and 3-ethyl-5-(2-ethylbutyl) octadecane [25]. Tetradecane, 2,4,4-trimethylpent-2-ene, and 2-methylundecane were found in higher concentrations than the rest of the aromatic compounds in this group and would accordingly participate to a greater extent in the formation of the taste and aroma profiles of the obtained fermented milk. The largest amount of 2,4,4-trimethylpent-2-ene was produced by the KM3 starter culture, followed by the KM1 starter culture. Lower and similar concentrations of this metabolite were found in milk fermented with KM2 and KM4. 2-methyluneucan in higher and similar concentrations was found in yogurts obtained with starter cultures KM2 and KM3. Lower and also comparable concentrations of this metabolite were found in yogurts fermented with KM2 and KM4. The aromatic compound undecane was not detected in milk fermented with a starter culture KM2. This metabolic component was found in different concentrations in milk fermented with other starter cultures. It is noteworthy that only in starter culture KM4 was a significantly high concentration of hexadecane compared to the milk obtained with other cultures. The other aromatic components of this group, 3-carne, tridecane, 2,2,4,6,6-pentamethylhept-3-ene, nonadecane, pentadecane, 3-ethyl-5-(2-ethylbutyl)octadecane, and 2,4,6-trimethyldecane, were found in significantly lower concentrations.

The obtained volatile compound profiles for the designed starter cultures clearly showed that the composition of each combination affected the aroma of the produced yogurts. Dan et al. reported similar findings in an investigation of milk fermented with different combinations of *L. bulgaricus* and *S. thermophilus* [15]. Our results are also in line with the conclusions of Ott et al. [29], Zheng et al. [30], and Papaionnau et al. [31], who found that the type of starter culture influences the volatile compound profile of yogurt. Duran et al. compared the aroma-forming metabolite profiles of three strains for kefir production [32] and also found significant differences. Other authors have studied the effect of a single species on the fermentation process rather than the variation of aromatic metabolites between different starters [33,34]. Such profiles can also be used for quality control of the fermentation process, as suggested by Zaręba et al. [35].

It is essential that all starter cultures retain their ability to produce the main aromatic components after the lyophilization process, which is largely determined by sublimation drying regimes. This is especially true if the starters are to be used for direct application. With this reason in mind, yogurts were prepared with production (liquid) starter cultures and lyophilized cultures by the method of direct application. A comparison was made with respect to their metabolic profiles. The results of these studies are presented in Table 3.

From the data presented in Table 3, it is evident that all metabolic components in the liquid starter cultures were characterized by larger relative areas than in the lyophilized ones, which shows that in milk obtained with liquid cultures, metabolic components will be in higher concentrations. The preparation and lyophilization of the starter cultures did not affect the ability of the starter cultures to synthesize aromatics from the aldehyde group. However, in the group of ketones, the effect of freeze-drying was observed in the ability of starter cultures KM1, KM3, and KM4 to produce 2-nonanone. In yogurts obtained with lyophilized cultures by the direct feed method, this metabolic component was not detected compared to milks fermented with liquid starter cultures. A similar trend was observed in the group of organic acids. Butyric acid was not detected in yogurts obtained with lyophilized starter cultures KM2, KM3, and KM4, while in milk fermented with liquid cultures, butyric acid was detected. Starter culture KM1 in lyophilized state lost its ability to produce 1,2-benzenedicarboxylic acid compared to its liquid form.

The process of freeze-drying did not affect the ability of the starter cultures to produce all aromatic alcoholic compounds (Table 3). In the ester components group, it was observed that only lyophilized KM1 did not synthesize tridec-2-ynyl 3-methylbut-2-enoate and 1-(2,4-dimethylphenyl)ethanone. There was a loss of ability to synthesize the metabolites 3-carne and 2,2,4,6,6-pentamethylhept-3-ene from lyophilized starter cultures KM1, KM2, and KM4 compared to liquid in the aromatic hydrocarbons group. Tridecane was not synthesized only by the lyophilized KM2 starter culture. 2,4,6-Trimethyldecane was not detected in milk fermented with lyophilized starter culture KM1. Pentadecane was absent in yogurts obtained with lyophilized starter cultures KM2 and KM4. However, 3-ethyl-5-(2-ethylbutyl)-octadecane was not detected only in milk fermented with the lyophilized KM1 starter culture.

In conclusion, the lyophilized starter culture KM1 did not produce a total of eight different metabolites, KM2 and KM4 did not synthesize a total of five metabolites, and KM3 did not synthesize a total of three metabolites. Since the structure of DNA is maintained by water in a quasi-crystalline state, the dehydration that occurs during freeze-drying clearly affected the structure of the genes responsible for the production of said metabolic products. The effect of freeze-drying on the DNA molecule of lactic acid bacteria is well established [36,37,38]. From the conducted studies, it can be concluded that the microorganisms in starter culture KM3 had the highest genetic resistance in terms of maintaining the ability to produce most metabolites. The greatest effect of cell dehydration on the structure of the genes responsible for the production of certain metabolites was observed in KM1. Starter cultures KM2 and KM4 were intermediate in terms of preserving their ability to produce certain aromatic components.

### 3.3. Effect of the Pre-Cooling Stage on the Volatile Compound Profiles of the Starter Cultures

One of the obligatory stages in the production of traditional Bulgarian yogurt is pre-cooling; thus, its influence on the aromatic metabolic profiles of the obtained cultures was studied. The pre-cooling of the coagulated milk took 25 min. The results of these studies are presented in Table 4. Pre-cooling did not significantly affect the metabolic components. In all studied starters, the aromatic metabolites were characterized by similar relative peak areas for each compound in yogurt with and without pre-cooling. In the aldehyde group, pre-cooling affected only the production of 2-octenal by starter culture KM2. This compound was not found in yogurt which was obtained without pre-cooling. A similar trend was observed in the ketone group. In yogurt fermented with KM3 without pre-cooling, 2-nonanone was not detected compared to milk obtained with pre-cooling. 3-Methyl-2-butanone was not detected in yogurts obtained with starter cultures KM2, KM3, and KM4 without pre-cooling.

The process of pre-cooling did not significantly affect the production of organic acids and aromatic alcohols by the studied starter cultures. In the ester components group, pre-cooling had a significant effect on the formation of the ethyl propanoate and 2-methyl-2H-furan-5-one when using a KM2 starter culture. In products that were not subjected to pre-cooling, these metabolites were not synthesized. In the aromatic hydrocarbons group, pre-cooling affected the formation of 3-carne from starter culture KM3 and pentadecane produced from starter cultures KM2 and KM4. In yogurt obtained with those starters without pre-cooling, the mentioned metabolites were not detected.

From this study, it can be concluded that the formation of aromatic metabolic products from KM1 was not significantly affected by the process of pre-cooling. However, in the production of yogurts, it is necessary to cool the coagulated milk for 25 min, which would allow for the production of the full range of metabolic products, forming the organoleptic profile of the yogurt.

### 3.4. Influence of the Fat Content of Milk on the Volatile Compound Profiles of the Starter Cultures

In a series of experiments, the influence of the fat content of the milk substrate on the metabolic profile of the starter cultures was studied. Homogenized and fat-standardized raw cow’s milk with 0.1%, 1.5%, and 3.0% crude fat was used for these experiments. The results of these studies are presented in Table 5.

The data show that the main aromatic component from the aldehyde group in these yogurts was benzaldehyde. It was found in the largest amounts in the milk fermented with starter culture KM3, and increasing the fat content of the milk increased the benzaledyde concentration. At 3.0% fat content, the concentration of this metabolite had the highest relative peak area. In the case of milk with 1.5% fat content, its concentration was lower, and the lowest concentration was detected in skimmed milk (fat content 0.1%). A similar trend was observed for benzaldehyde in starter cultures KM1 and KM4. Yogurt obtained from milk with 3.0% fat content and starter culture KM4 also had the highest concentration of benzaldehyde, which was close to that of KM3 with 3.0% fat content. In the production of milk with KM4 with 1.5% and 0.1% fat content, a significant decrease in the concentration of benzaldehyde was observed. The next aldehydes component that was in higher concentrations than the rest was acetaldehyde. The highest concentration of this component was observed in milk with 1.5% fat content and starter cultures KM1, KM3, and KM4. In starter cultures KM3 and KM4, lower concentrations of this metabolite were detected in 3.0% fat milk, followed by skimmed milk. In starter culture KM1 with 3.0% fat content, the lowest concentration of acetaldehyde was observed. Skimmed milk occupied an intermediate place in the concentration of this metabolite. In milk fermented with starter culture KM2 with 3.0% and 1.5% fat content, the highest concentrations of acetaldehyde were observed. The other components of the aldehyde group were in lower concentrations which increased with increases in milk fat content.

From the ketone group, 2-pentanone, acetoin, 2,3-butanedione, and 2-nonanone had the highest concentrations of aromatic components and respectively participated to a greater extent in the formation of the organoleptic profiles of yogurts. In the case of starter culture KM1, increases in milk fat content increased 2-pentanone concentrations. A similar trend was observed in yogurts obtained with KM2. Milk fermented with KM3 and KM4 with 1.5% and 3.0% fat content, respectively, produced very similar concentrations of 2-pentanone, which showed that a further increase in milk fat content above 1.5% did not significantly affect the synthesis of this component by this starter culture. However, in skimmed milk fermented with KM4, the concentration of 2-pentanone was significantly lower. Regarding the synthesis of acetoin, it was evident that in milk fermented with starter culture KM4, the fat content did not affect the concentration of this metabolite. In starter culture KM1, similar values were detected for concentrations of acetoin at 1.5% and 3.0% fat content. This shows that an increase in fat content above 1.5% did not significantly affect acetoin synthesis. A similar trend was observed for starter culture KM3. Only in starter culture KM2 was there a continuous increase in the concentration of acetoin and milk with a fat content of 3.0%, the relative peak area for this component was 0.8701.

The fat content of milk affected the synthesis of 2,3-butanedione in starter cultures KM1, KM2, and KM3; the highest concentration of this metabolite was detected in milk with 3.0% fat content. In milk fermented with starter culture KM4, fat content above 1.5% did not significantly affect the synthesis of this metabolite. The other aromatic components of the ketone group were in lower concentrations and increases in fat content did not significantly affect their amount. Regarding the influence of fat content on the synthesis of organic acids, increases in milk fat content above 1.5% did not have a significant effect on the amounts of acids formed, except for the production of hexanoic acid in milk fermented with starter cultures KM2 and KM3, where a continuous increase in the concentration of this acid was detected with increases in fat content, and the highest concentration of this metabolite was observed in milk with 3.0% fat content. For the alcohol components, increases in the concentrations of 2-furanmethanol were observed with increases in the milk fat content of all four starter cultures. A similar trend was observed for 3-methyl-2-butanol in starter culture KM2, in which the highest concentration of this metabolite was observed in milk with 3.0% fat content, relative peak area 0.4164. The amount of 3-methyl-2-butanol in other starter cultures was not significantly affected by increases in milk fat content above 1.5%. A similar trend was observed for the other aromatic alcohols.

The ester components and aromatic hydrocarbons concentrations were not significantly affected by increases in milk fat content in the milk fermented with the selected starter cultures.

In general, when determining the metabolic profiles of the symbiotic starter cultures, the presence of new ester components which were absent in monocultures of lactobacilli and streptococci—ethyl ester of propionic acid, trideca-2-ylyl ester of 3-methyl-2-butanoic acid, and 1(2,4-dimethylphenyl)-ethanone—were detected [25]. New aromatic hydrocarbons—2,4,4,-trimethyl-2-pentene, nonadecane, and 3-ethyl-5-(2-ethylbutyl)-octadecane—were also synthesized by the starters.

Lipid metabolism is one of the pathways for aromatic metabolite synthesis by the starter cultures in yogurt [1]. Few studies have investigated the influence of milk fat content on the volatile compound profiles of yogurt [39,40]. Bao et al. observed increases in ketones and carbonyl acid concentrations with increases in free fatty acid content in milk fermented by *Lactobacillus casei* GBHM-21 [41]. Our results suggest that the studied starter cultures also increase the synthesis of volatile compounds when the crude milk fat is increased from 0.1 to 1.5%. However, there was no significant difference in most metabolite concentrations between milk with fat contents 1.5 and 3%.

## 4. Conclusions

In the present study, a method was developed for the design and characterisation of starter cultures for Bulgarian yogurt. Complete and stable symbiosis between *L. bulgaricus* and *S. thermophilus* was confirmed only for combinations where the ratio of lactobacilli to streptococci was approximately 1:3, 1:9, or 1:5 in favor of streptococci. Stable symbiotic starter cultures were established and their aromatic-metabolic profiles were determined, demonstrating the specificity of each combination depending on the ratios of the monocultures. Starter cultures KM4 and KM2 were found to be particularly suitable for obtaining functional lactic acid products as they synthesized the largest amounts of butyric acid, which has highly pronounced health benefits.

It has been shown that freeze-drying has a significant effect on the aroma-forming ability of the selected starter cultures, with KM3 having the highest genetic resistance to freeze-drying in terms of producing aromatic components.

With regard to a technological parameter such as pre-cooling, in the production of yogurt, it is necessary to pre-cool coagulated milk for 25 min, during which time it is possible to more fully develop the range of metabolic products that form the taste and aroma of fermented milk.

It was also found that increases in the fat content of milk in the range of 1.5 to 3.0% did not significantly affect the levels of the majority of aroma components synthesized by the selected starter cultures.

The determined aromatic-metabolic profiles of the starter cultures can be used to prove and assess the authenticity of traditional Bulgarian dairy products. The same approach could be applied to the design of starter cultures for other fermented foods with specific volatile compound profiles.

## Figures and Tables

**Table 1:** Biocompatibility of the *Lactobacillus delbrueckii* subsp. *bulgaricus* and *Streptococcus salivarius* subsp. *thermophilus* strains.

Tested Strain	*L. bulgaricus* MG1	*L. bulgaricus* MG2	*L. bulgaricus* MG3	*L. bulgaricus* MG4	*L. bulgaricus* MG5	*L. bulgaricus* MG6	*L. bulgaricus* MG7	*L. bulgaricus* MG8	*L. bulgaricus* MG9
*S. thermophilus* T1	−	−	+	−	−	−	+	−	±
*S. thermophilus* T2	−	−	+	−	−	−	−	−	−
*S. thermophilus* T3	−	−	−	−	−	−	−	−	−
*S. thermophilus* T4	−	−	−	−	−	−	−	−	−
*S*. *thermophilus* T5	−	−	−	−	−	−	−	−	−
*S*. *thermophilus* T6	−	−	−	−	−	−	±	−	±
*S*. *thermophilus* T7	−	−	−	−	−	−	−	−	−
*S*. *thermophilus* T8	−	−	−	−	−	−	−	−	−

+—biocompatible strains; −—biononcompatible strain; ±—borderline biocompatibility.

**Table 2:** Aromatic components produced by symbiotic starter cultures KM1, KM2, KM3, and KM4.

Metabolic Profile of the Starter Cultures							
No.	Volatile Compound	Chemical Formula	RT (min)	Starter Culture *			
				KM1	KM2	KM3	KM4
Aldehyde Compounds							
1	Acetaldehyde	C_2_H_4_O	1.89	0.3075	0.2000	0.2231	0.0944
2	Furaldehyde	C_5_H_4_O_2_	9.53	0.1076	0.0893	0.0593	0.0942
3	3-Hydroxybutanal	C_4_H_8_O_2_	13.23	0.0216	0.0102	0.0144	0.0096
4	Benzaldehyde	C_7_H_6_O	15.22	1.0967	1.0695	1.2591	1.1134
5	Benzacetaldehyde	C_8_H_8_O	18.65	0.0150	0.0041	0.0054	0.0089
6	Ethylbenzaldehyde	C_9_H_10_O	23.97	0.0152	0.0104	0.0120	0.0162
7	2-Octenal	C_8_H_14_O	29.3	0.0499	0.0266	0.0484	0.0343
8	Decanal	C_10_H_20_O	40.91	ND	ND	ND	0.0084
Ketone Compounds							
9	2-Pentanone	C_5_H_10_O	2.52	0.6280	0.4769	0.6371	0.1781
10	Acetoin	C_4_H_8_O_2_	4.89	0.7122	0.5955	0.6422	0.9376
11	2,3-Butanedione	C_4_H_6_O_2_	11.86	0.1624	0.1408	0.1609	0.1588
12	2-Acetylfuran	C_6_H_6_O_2_	12.88	0.0064	0.0087	0.0029	0.0083
13	2-Nonanone	C_9_H_18_O	20.3	0.1164	ND	0.1237	0.1242
14	2-Heptanone	C_7_H_14_O	24.13	0.0172	0.0132	0.0234	0.0148
15	3-Methyl-2-butanone	C_5_H_10_O	27.43	ND	0.0022	0.0034	0.0030
16	2-Undecanone	C_11_H_22_O	27.73	0.0377	0.0266	0.0500	0.0300
Acid Compounds							
17	Formic acid	CH_2_O_2_	3.79	0.0304	0.0219	0.0338	0.1109
18	Butyric acid	C_4_H_8_O_2_	7.54	0.0060	0.0093	0.0031	0.0102
19	Acetic acid	C_2_H_4_O_2_	14.21	0.2068	0.1990	0.2415	0.2066
20	Hexanoic acid	C_6_H_12_O_2_	15.88	0.1380	0.1258	0.1406	0.2632
21	Pentanoic acid	C_5_H_10_O_2_	16.35	0.0057	0.0060	0.0189	0.0102
22	Benzoic acid	C_7_H_6_O_2_	23.08	0.0157	0.0119	0.0159	0.0190
23	Octanoic acid	C_8_H_16_O_2_	23.29	0.0151	0.0143	0.0186	0.0174
24	1,2-Benzenedicarboxylic acid	C_8_H_6_O_4_	28.82	0.0269	0.0187	0.0317	0.0196
Alcohol Compounds							
25	2-Furanmethanol	C_5_H_6_O_2_	10.32	0.4998	0.4663	0.5328	0.5452
26	Ethanol, 2-(octyloxy)-	C_10_H_22_O_2_	19.17	0.1142	0.0835	0.1234	0.0793
27	3-Methyl-2-butanol	C_5_H_12_O	20.06	0.2526	0.1043	0.3017	0.2572
28	2-Undecanol	C_11_H_24_O	28.04	0.0086	0.0031	0.0197	0.0028
Ester Compounds							
29	Propanoic acid, ethenyl ester	C_5_H_8_O_2_	4.09	0.0129	0.0330	0.0504	0.0327
30	2(5H)-Furanone, 5-methyl-	C_5_H_6_O_2_	11.22	ND	0.0047	ND	ND
31	Benzoic acid, 2-ethylhexyl ester	C_15_H_22_O_2_	19.55	0.0227	0.0178	0.0251	0.0262
32	3-Methyl-2-butenoic acid, tridec-2-ynyl ester	C_18_H_30_O_2_	28.86	0.0105	0.0042	0.0171	0.0032
33	Ethanone, 1-(2,4-dimethylphenyl)-	C_10_H_12_O	29.65	0.0196	0.0171	0.0448	0.0156
34	4-Ethylbenzoic acid, methyl ester	C_10_H_12_O_2_	29.79	0.0224	0.0180	0.0260	0.0173
Aromatic Hydrocarbons							
35	3-Carene	C_10_H_16_	13.45	0.0038	0.0035	0.0022	0.0076
36	Undecane	C_11_H_24_	18.52	0.0067	ND	0.0051	0.0091
37	Tridecane	C_13_H_28_	26.76	0.0035	0.0036	0.0040	0.0053
38	3-Heptene, 2,2,4,6,6-pentamethyl-	C_12_H_24_	28.18	0.0023	0.0017	0.0074	0.0032
39	2-Methylundecane	C_12_H_26_	28.39	0.0269	0.0187	0.0317	0.0196
40	2-Pentene, 2,4,4-trimethyl	C_8_H_16_	29.22	0.0338	0.0114	0.0646	0.0106
41	Tetradecane	C_14_H_30_	31.12	0.0415	0.0393	0.0500	0.0448
42	2,4,6-Trimethyldecane	C_13_H_28_	31.21	0.0032	0.0023	0.0055	0.0028
43	Nonadecane	C_19_H_40_	34.9	0.0080	0.0074	0.0092	0.0097
44	Pentadecane	C_15_H_32_	35.02	0.0038	0.0021	0.0029	0.0017
45	Hexadecane	C_16_H_34_	39.02	0.0045	0.0045	0.0040	0.0139
46	Octadecane, 3-ethyl-5-(2-ethylbutyl)	C_26_H_54_	40.21	0.0020	0.0039	0.0048	0.0040
47	Octadecane	C_18_H_38_	41.1	0.0093	0.0083	0.0092	0.0096

* Relative peak area against an internal standard. ND—the compound was not detected.

**Table 3:** Aromatic components produced from liquid and lyophilized symbiotic starter cultures KM1, KM2, KM3, and KM4.

Metabolic Profile of the Starter Cultures (Liquid and Lyophilized)											
No.	Volatile Compound	Chemical Formula	RT (min)	Starter Culture *							
				KM1 Liquid	KM1 Lyophilized	KM2 Liquid	KM2 Lyophilized	KM3 Liquid	KM3 Lyophilized	KM4 Liquid	KM4 Lyophilized
Aldehyde Compounds											
1	Acetaldehyde	C_2_H_4_O	1.89	0.3075	0.1919	0.2000	0.1206	0.2231	0.1767	0.0944	0.0361
2	Furaldehyde	C_5_H_4_O_2_	9.53	0.1076	0.0739	0.0893	0.0610	0.0593	0.0436	0.0942	0.0810
3	3-Hydroxybutanal	C_4_H_8_O_2_	13.23	0.0216	0.0172	0.0102	0.0085	0.0144	0.0126	0.0096	0.0066
4	Benzaldehyde	C_7_H_6_O	15.22	1.0967	0.8991	1.0695	1.1038	1.2591	1.1697	1.1134	1.0092
5	Benzacetaldehyde	C_8_H_8_O	18.65	0.0150	0.0100	0.0041	0.0038	0.0054	0.0036	0.0089	0.063
6	Ethylbenzaldehyde	C_9_H_10_O	23.97	0.0152	0.0125	0.0104	0.0070	0.0120	0.0011	0.0162	0.0021
7	2-Octenal	C_8_H_14_O	29.3	0.0499	0.0496	0.0266	0.0206	0.0484	0.0166	0.0343	0.0149
8	Decanal	C_10_H_20_O	40.91	ND	ND	ND	ND	ND	ND	0.0084	0.0053
Ketone Compounds											
9	2-Pentanone	C_5_H_10_O	2.52	0.6280	0.4357	0.4769	0.3342	0.6371	0.6208	0.1781	0.1363
10	Acetoin	C_4_H_8_O_2_	4.89	0.7122	0.5421	0.5955	0.3088	0.6422	0.5043	0.9376	0.4762
11	2,3-Butanedione	C_4_H_6_O_2_	11.86	0.1624	0.1003	0.1408	0.1215	0.1609	0.1346	0.1588	0.1322
12	2-Acetylfuran	C_6_H_6_O_2_	12.88	0.0064	0.0045	0.0087	0.0036	0.0029	0.0026	0.0083	0.0042
13	2-Nonanone	C_9_H_18_O	20.3	0.1164	ND	ND	ND	0.1237	ND	0.1242	ND
14	2-Heptanone	C_7_H_14_O	24.13	0.0172	0.0141	0.0132	0.0117	0.0234	0.0217	0.0148	0.0113
15	3-Methyl-2-butanone	C_5_H_10_O	27.43	ND	ND	0.0022	0.0021	0.0034	0.0032	0.0030	0.0024
16	2-Undecanone	C_11_H_22_O	27.73	0.0377	0.0349	0.0266	0.0203	0.0500	0.0430	0.0300	0.0251
Acid Compounds											
17	Formic acid	CH_2_O_2_	3.79	0.0304	0.0090	0.0219	0.0084	0.0338	0.0206	0.1109	0.0294
18	Butyric acid	C_4_H_8_O_2_	7.54	0.0060	0.0017	0.0093	ND	0.0031	ND	0.0102	ND
19	Acetic acid	C_2_H_4_O_2_	14.21	0.2068	0.1298	0.1990	0.1500	0.2415	0.1847	0.2066	0.1995
20	Hexanoic acid	C_6_H_12_O_2_	15.88	0.1380	0.1091	0.1258	0.0601	0.1406	0.1257	0.2632	0.1387
21	Pentanoic acid	C_5_H_10_O_2_	16.35	0.0057	0.0018	0.0060	0.0022	0.0189	0.0112	0.0102	0.0045
22	Benzoic acid	C_7_H_6_O_2_	23.08	0.0157	0.0050	0.0119	0.0019	0.0159	0.0076	0.0190	0.0112
23	Octanoic acid	C_8_H_16_O_2_	23.29	0.0151	0.0105	0.0143	0.0071	0.0186	0.0148	0.0174	0.0147
24	1,2-Benzenedicarboxylic acid	C_8_H_6_O_4_	28.82	0.0269	ND	0.0187	0.0048	0.0317	0.0156	0.0196	0.0137
Alcohol Compounds											
25	2-Furanmethanol	C_5_H_6_O_2_	10.32	0.4998	0.3922	0.4663	0.3549	0.5328	0.3863	0.5452	0.3649
26	Ethanol, 2-(octyloxy)-	C_10_H_22_O_2_	19.17	0.1142	0.0562	0.0835	0.0621	0.1234	0.0928	0.0793	0.0743
27	3-Methyl-2-butanol	C_5_H_12_O	20.06	0.2526	0.1267	0.1043	0.0836	0.3017	0.2603	0.2572	0.1916
28	2-Undecanol	C_11_H_24_O	28.04	0.0086	0.0021	0.0031	0.0027	0.0097	0.0097	0.0028	0.0018
Ester Compounds											
29	Propanoic acid, ethenyl ester	C_5_H_8_O_2_	4.09	0.0129	0.0045	0.0330	0.0103	0.0504	0.0427	0.0327	0.0061
30	2(5H)-Furanone, 5-methyl-	C_5_H_6_O_2_	11.22	ND	ND	0.0047	0.0032	ND	ND	ND	ND
31	Benzoic acid, 2-ethylhexyl ester	C_15_H_22_O_2_	19.55	0.0227	0.0171	0.0178	0.0132	0.0251	0.0209	0.0262	0.0253
32	3-Methyl-2-butenoic acid, tridec-2-ynyl ester	C_18_H_30_O_2_	28.86	0.0105	ND	0.0042	0.0028	0.0171	0.0156	0.0032	0.0023
33	Ethanone, 1-(2,4-dimethylphenyl)-	C_10_H_12_O	29.65	0.0196	ND	0.0171	0.0155	0.0448	0.0398	0.0156	0.0078
34	4-Ethylbenzoic acid, methyl ester	C_10_H_12_O_2_	29.79	0.0224	0.0159	0.0180	0.0108	0.0260	0.0200	0.0173	0.0147
Aromatic Hydrocarbons											
35	3-Carene	C_10_H_16_	13.45	0.0038	ND	0.0035	ND	0.0022	0.0017	0.0076	ND
36	Undecane	C_11_H_24_	18.52	0.0067	0.0031	ND	ND	0.0051	0.0040	0.0091	0.0056
37	Tridecane	C_13_H_28_	26.76	0.0035	0.0036	0.0036	ND	0.0040	0.0040	0.0053	0.0034
38	3-Heptene, 2,2,4,6,6-pentamethyl-	C_12_H_24_	28.18	0.0023	ND	0.0017	ND	0.0074	ND	0.0032	ND
39	2-Methylundecane	C_12_H_26_	28.39	0.0269	0.0134	0.0187	0.0060	0.0317	0.0157	0.0196	0.0142
40	2-Pentene, 2,4,4-trimethyl	C_8_H_16_	29.22	0.0338	0.0128	0.0114	0.0089	0.0646	0.0442	0.0106	0.0095
41	Tetradecane	C_14_H_30_	31.12	0.0415	0.0377	0.0393	0.0257	0.0500	0.0505	0.0448	0.0486
42	2,4,6-Trimethyldecane	C_13_H_28_	31.21	0.0032	ND	0.0023	0.0017	0.0055	0.0046	0.0028	0.0027
43	Nonadecane	C_19_H_40_	34.9	0.0080	0.0072	0.0074	0.0035	0.0092	0.0074	0.0097	0.0075
44	Pentadecane	C_15_H_32_	35.02	0.0038	0.0028	0.0021	ND	0.0029	0.0016	0.0017	ND
45	Hexadecane	C_16_H_34_	39.02	0.0045	0.036	0.0045	0.0031	0.0040	0.0026	0.0139	0.0057
46	Octadecane, 3-ethyl-5-(2-ethylbutyl)	C_26_H_54_	40.21	0.0020	ND	0.0039	0.0026	0.0048	0.0037	0.0040	0.0031
47	Octadecane	C_18_H_38_	41.1	0.0093	0.0055	0.0083	0.0075	0.0092	0.0064	0.0096	0.0086

* Relative peak area against an internal standard. ND—the compound was not detected.

**Table 4:** Influence of the pre-cooling process on the metabolic profiles of symbiotic starter cultures KM1, KM2, KM3, and KM4.

Metabolic Profiles of the Starter Cultures with or without Pre-Cooling											
No.	Volatile Compound	Chemical Formula	RT (min)	Starter Culture *							
				KM1+	KM1−	KM2+	KM2−	KM3+	KM3−	KM4+	KM4−
Aldehyde Compounds											
1	Acetaldehyde	C_2_H_4_O	1.89	0.3075	0.2471	0.2000	0.0719	0.2231	0.1872	0.0944	0.0854
2	Furaldehyde	C_5_H_4_O_2_	9.53	0.1076	0.1006	0.0893	0.0802	0.0593	0.0529	0.0942	0.0669
3	3-Hydroxybutanal	C_4_H_8_O_2_	13.23	0.0216	0.0156	0.0102	0.0065	0.0144	0.0142	0.0096	0.0055
4	Benzaldehyde	C_7_H_6_O	15.22	1.0967	1.0422	1.0695	1.0291	1.2591	1.1197	1.1134	1.0274
5	Benzacetaldehyde	C_8_H_8_O	18.65	0.0150	0.0105	0.0041	0.0032	0.0054	0.0040	0.0089	0.0075
6	Ethylbenzaldehyde	C_9_H_10_O	23.97	0.0152	0.0139	0.0104	ND	0.0120	0.0104	0.0162	0.0143
7	2-Octenal	C_8_H_14_O	29.3	0.0499	0.0446	0.0266	0.0182	0.0484	0.0435	0.0343	0.0340
8	Decanal	C_10_H_20_O	40.91	ND	ND	ND	ND	ND	ND	0.0084	0.0025
Ketone Compounds											
9	2-Pentanone	C_5_H_10_O	2.52	0.6280	0.5034	0.4769	0.3231	0.6371	0.6211	0.1781	0.1041
10	Acetoin	C_4_H_8_O_2_	4.89	0.7122	0.6191	0.5955	0.5628	0.6422	0.6238	0.9376	0.5897
11	2,3-Butanedione	C_4_H_6_O_2_	11.86	0.1624	0.1212	0.1408	0.1340	0.1609	0.1598	0.1588	0.1496
12	2-Acetylfuran	C_6_H_6_O_2_	12.88	0.0064	0.0021	0.0087	0.0079	0.0029	0.0024	0.0083	0.0079
13	2-Nonanone	C_9_H_18_O	20.3	0.1164	0.1062	ND	ND	0.1237	ND	0.1242	0.1002
14	2-Heptanone	C_7_H_14_O	24.13	0.0172	0.0144	0.0132	0.0090	0.0234	0.0198	0.0148	0.0125
15	3-Methyl-2-butanone	C_5_H_10_O	27.43	ND	ND	0.0022	ND	0.0034	ND	0.0030	ND
16	2-Undecanone	C_11_H_22_O	27.73	0.0377	0.0281	0.0266	0.0207	0.0500	0.0374	0.0300	0.0302
Acid Compounds											
17	Formic acid	CH_2_O_2_	3.79	0.0304	0.0773	0.0219	0.0228	0.0338	0.0370	0.1109	0.0392
18	Butyric acid	C_4_H_8_O_2_	7.54	0.0060	0.0059	0.0093	0.0024	0.0031	0.0030	0.0102	0.0048
19	Acetic acid	C_2_H_4_O_2_	14.21	0.2068	0.2377	0.1990	0.1854	0.2415	0.2593	0.2066	0.1940
20	Hexanoic acid	C_6_H_12_O_2_	15.88	0.1380	0.1224	0.1258	0.0539	0.1406	0.1417	0.2632	0.2537
21	Pentanoic acid	C_5_H_10_O_2_	16.35	0.0057	0.0048	0.0060	0.0053	0.0189	0.0129	0.0102	0.0086
22	Benzoic acid	C_7_H_6_O_2_	23.08	0.0157	0.0134	0.0119	0.0126	0.0159	0.0164	0.0190	0.0157
23	Octanoic acid	C_8_H_16_O_2_	23.29	0.0151	0.0144	0.0143	0.0120	0.0186	0.0190	0.0174	0.0168
24	1,2-Benzenedicarboxylic acid	C_8_H_6_O_4_	28.82	0.0269	0.0259	0.0187	0.0137	0.0317	0.0261	0.0196	0.0184
Alcohol Compounds											
25	2-Furanmethanol	C_5_H_6_O_2_	10.32	0.4998	0.4177	0.4663	0.4277	0.5328	0.5350	0.5452	0.5172
26	Ethanol, 2-(octyloxy)-	C_10_H_22_O_2_	19.17	0.1142	0.0191	0.0835	0.1005	0.1234	0.1106	0.0793	0.0771
27	3-Methyl-2-butanol	C_5_H_12_O	20.06	0.2526	0.2174	0.1043	0.0890	0.3017	0.2376	0.2572	0.2453
28	2-Undecanol	C_11_H_24_O	28.04	0.0086	0.0081	0.0031	0.0091	0.0097	0.0055	0.0028	0.0029
Ester Compounds											
29	Propanoic acid, ethenyl ester	C_5_H_8_O_2_	4.09	0.0129	0.0653	0.0330	ND	0.0504	0.0256	0.0327	0.0275
30	2(5H)-Furanone, 5-methyl-	C_5_H_6_O_2_	11.22	ND	ND	0.0047	ND	ND	ND	ND	ND
31	Benzoic acid, 2-ethylhexyl ester	C_15_H_22_O_2_	19.55	0.0227	0.0204	0.0178	0.0136	0.0251	0.0235	0.0262	0.0260
32	3-Methyl-2-butenoic acid, tridec-2-ynyl ester	C_18_H_30_O_2_	28.86	0.0105	0.0083	0.0042	0.0031	0.0171	0.0092	0.0032	0.0040
33	Ethanone, 1-(2,4-dimethylphenyl)-	C_10_H_12_O	29.65	0.0196	0.0172	0.0171	0.0165	0.0448	0.0298	0.0156	0.0155
34	4-Ethylbenzoic acid, methyl ester	C_10_H_12_O_2_	29.79	0.0224	0.0203	0.0180	0.0152	0.0260	0.0233	0.0173	0.0169
Aromatic Hydrocarbons											
35	3-Carene	C_10_H_16_	13.45	0.0038	0.0014	0.0035	0.0010	0.0022	ND	0.0076	0.0071
36	Undecane	C_11_H_24_	18.52	0.0067	0.0059	ND	ND	0.0051	0.0045	0.0091	0.0088
37	Tridecane	C_13_H_28_	26.76	0.0035	0.0033	0.0036	0.0036	0.0040	0.0039	0.0053	0.0042
38	3-Heptene, 2,2,4,6,6-pentamethyl-	C_12_H_24_	28.18	0.0023	0.0022	0.0017	0.0019	0.0074	0.0034	0.0032	0.0035
39	2-Methylundecane	C_12_H_26_	28.39	0.0269	0.0053	0.0187	0.0068	0.0317	0.0044	0.0196	0.0012
40	2-Pentene, 2,4,4-trimethyl	C_8_H_16_	29.22	0.0338	0.0229	0.0114	0.0099	0.0646	0.0213	0.0106	0.0059
41	Tetradecane	C_14_H_30_	31.12	0.0415	0.0338	0.0393	0.0402	0.0500	0.0529	0.0448	0.0419
42	2,4,6-Trimethyldecane	C_13_H_28_	31.21	0.0032	0.0039	0.0023	0.0038	0.0055	0.0039	0.0028	0.0033
43	Nonadecane	C_19_H_40_	34.9	0.0080	0.0069	0.0074	0.0070	0.0092	0.0092	0.0097	0.0088
44	Pentadecane	C_15_H_32_	35.02	0.0038	0.0036	0.0021	ND	0.0029	0.0023	0.0017	ND
45	Hexadecane	C_16_H_34_	39.02	0.0045	0.0039	0.0045	0.0039	0.0040	0.0033	0.0139	0.0042
46	Octadecane, 3-ethyl-5-(2-ethylbutyl)	C_26_H_54_	40.21	0.0020	0.0020	0.0039	0.0031	0.0048	0.0047	0.0040	0.0024
47	Octadecane	C_18_H_38_	41.1	0.0093	0.0091	0.0083	0.0077	0.0092	0.0088	0.0096	0.0074

* Relative peak area against an internal standard; +—with pre-cooling; −—without pre-cooling. ND—the compound was not detected.

**Table 5:** Influence of crude fat content on the metabolic profiles of symbiotic starter cultures KM1, KM2, KM3, and KM4.

Metabolic Profiles of the Starter Cultures at Different Crude Fat Contents															
No.	Volatile Compound	Chemical Formula	RT (min)	Starter Culture *											
				KM1			KM2			KM3			KM4		
				0.1%	1.5%	3%	0.1%	1.5%	3%	0.1%	1.5%	3%	0.1%	1.5%	3%
Aldehyde Compounds															
1	Acetaldehyde	C_2_H_4_O	1.89	0.3075	0.3956	0.2551	0.2000	0.1770	0.4361	0.2231	0.6182	0.4466	0.0944	0.4821	0.3514
2	Furaldehyde	C_5_H_4_O_2_	9.53	0.1076	0.1192	0.1536	0.0893	0.0928	0.1025	0.0593	0.0636	0.0760	0.0942	0.0995	0.1124
3	3-Hydroxybutanal	C_4_H_8_O_2_	13.23	0.0216	0.0234	0.0335	0.0102	0.0143	0.213	0.0144	0.0180	0.0202	0.0096	0.0121	0.0154
4	Benzaldehyde	C_7_H_6_O	15.22	1.0967	1.2824	1.3938	1.0695	1.2008	1.8039	1.2591	2.0811	2.5617	1.1134	1.5791	2.2774
5	Benzacetaldehyde	C_8_H_8_O	18.65	0.0150	0.0147	0.0135	0.0041	0.0034	0.0056	0.0054	0.0102	0.0092	0.0089	0.0081	0.0087
6	Ethylbenzaldehyde	C_9_H_10_O	23.97	0.0152	0.0211	0.0198	0.0104	0.0122	0.0141	0.0120	0.0138	0.0129	0.0162	0.0185	0.0192
7	2-Octenal	C_8_H_14_O	29.3	0.0499	0.0682	0.0745	0.0266	0.0364	0.1018	0.0484	0.1557	0.1616	0.0343	0.0463	0.0564
8	Decanal	C_10_H_20_O	40.91	ND	ND	ND	ND	ND	ND	ND	ND	ND	0.0084	0.0090	0.0106
Ketone Compounds															
9	2-Pentanone	C_5_H_10_O	2.52	0.6280	0.7872	0.8306	0.4769	0.6838	0.7701	0.6371	0.7808	0.7898	0.1781	0.5844	0.5773
10	Acetoin	C_4_H_8_O_2_	4.89	0.7122	0.9432	0.9029	0.5955	0.7883	0.8701	0.6422	0.7442	0.7996	0.9376	0.9914	0.9687
11	2,3-Butanedione	C_4_H_6_O_2_	11.86	0.1624	0.4123	0.6678	0.1408	0.2844	0.6021	0.1609	0.4795	1.0110	0.1588	0.5498	0.5963
12	2-Acetylfuran	C_6_H_6_O_2_	12.88	0.0064	0.0084	0.0090	0.0087	0.0102	0.0124	0.0029	0.0041	0.0048	0.0083	0.0092	0.0114
13	2-Nonanone	C_9_H_18_O	20.3	0.1164	0.1812	0.2542	ND	0.1341	0.2006	0.1237	0.3307	0.3619	0.1242	0.2580	0.2942
14	2-Heptanone	C_7_H_14_O	24.13	0.0172	0.0061	0.0105	0.0132	0.0047	0.0078	0.0234	0.0033	0.0101	0.0148	0.0167	0.0189
15	3-Methyl-2-butanone	C_5_H_10_O	27.43	ND	ND	ND	0.0022	0.0031	0.0022	0.0034	0.0033	0.0037	0.0030	0.0031	0.0025
16	2-Undecanone	C_11_H_22_O	27.73	0.0377	0.0305	0.0346	0.0266	0.0261	0.0306	0.0500	0.0617	0.0572	0.0300	0.0365	0.0369
Acid Compounds															
17	Formic acid	CH_2_O_2_	3.79	0.0304	0.0674	0.0938	0.0219	0.0272	0.0724	0.0338	0.0710	0.0900	0.1109	0.1672	0.2204
18	Butyric acid	C_4_H_8_O_2_	7.54	0.0060	0.0084	0.0113	0.0093	0.0127	0.0151	0.0031	0.0045	0.0066	0.0102	0.0126	0.0152
19	Acetic acid	C_2_H_4_O_2_	14.21	0.2068	0.2883	0.2973	0.1990	0.2372	0.2821	0.2415	0.4441	0.4157	0.2066	0.3332	0.2686
20	Hexanoic acid	C_6_H_12_O_2_	15.88	0.1380	0.2350	0.2005	0.1258	0.1682	0.4515	0.1406	0.4193	0.7167	0.2632	0.4023	0.4530
21	Pentanoic acid	C_5_H_10_O_2_	16.35	0.0057	0.0086	0.0121	0.0060	0.076	0.0078	0.0189	0.0196	0.0191	0.0102	0.0095	0.0090
22	Benzoic acid	C_7_H_6_O_2_	23.08	0.0157	0.0168	0.0146	0.0119	0.0176	0.0140	0.0159	0.0116	0.0104	0.0190	0.0114	0.0078
23	Octanoic acid	C_8_H_16_O_2_	23.29	0.0151	0.0138	0.0122	0.0143	0.0079	0.0109	0.0186	0.0121	0.0247	0.0174	0.0110	0.0168
24	1,2-Benzenedicarboxylic acid	C_8_H_6_O_4_	28.82	0.0269	0.0134	0.0145	0.0187	0.0156	0.0156	0.0317	0.0279	0.0215	0.0196	0.0317	0.0254
Alcohol Compounds															
25	2-Furanmethanol	C_5_H_6_O_2_	10.32	0.4998	0.5490	0.6410	0.4663	0.5300	0.6135	0.5328	0.5680	0.6154	0.5452	0.5680	0.6404
26	Ethanol, 2-(octyloxy)-	C_10_H_22_O_2_	19.17	0.1142	0.1173	0.1204	0.0835	0.0851	0.104	0.1234	0.2560	0.2894	0.0793	0.1004	0.1047
27	3-Methyl-2-butanol	C_5_H_12_O	20.06	0.2526	0.2542	0.2843	0.1043	0.2213	0.4164	0.3017	0.6576	0.5994	0.2572	0.2548	0.2483
28	2-Undecanol	C_11_H_24_O	28.04	0.0086	0.0025	0.0034	0.0031	0.0043	0.0025	0.0097	0.0114	0.0032	0.0028	0.054	0.0049
Ester Compounds															
29	Propanoic acid, ethenyl ester	C_5_H_8_O_2_	4.09	0.0129	0.0274	0.0447	0.0330	0.0721	0.0981	0.0504	0.0810	0.0683	0.0327	0.0672	0.0860
30	2(5H)-Furanone, 5-methyl-	C_5_H_6_O_2_	11.22	ND	ND	ND	0.0047	0.0052	0.0053	ND	ND	ND	ND	ND	ND
31	Benzoic acid, 2-ethylhexyl ester	C_15_H_22_O_2_	19.55	0.0227	0.0237	0.0264	0.0178	0.0175	0.0194	0.0251	0.0329	0.0329	0.0262	0.0249	0.0250
32	3-Methyl-2-butenoic acid, tridec-2-ynyl ester	C_18_H_30_O_2_	28.86	0.0105	0.0134	0.0145	0.0042	0.0156	0.0156	0.0171	0.0239	0.0215	0.0032	0.0117	0.0154
33	Ethanone, 1-(2,4-dimethylphenyl)-	C_10_H_12_O	29.65	0.0196	0.0204	0.0200	0.0171	0.0219	0.0199	0.0448	0.0487	0.0469	0.0156	0.0136	0.0148
34	4-Ethylbenzoic acid, methyl ester	C_10_H_12_O_2_	29.79	0.0224	0.0285	0.0290	0.0180	0.0178	0.0194	0.0260	0.0607	0.0588	0.0173	0.0292	0.0228
Aromatic Hydrocarbons															
35	3-Carene	C_10_H_16_	13.45	0.0038	0.0047	0.0067	0.0035	0.0041	0.0050	0.0022	0.0033	0.0050	0.0076	0.0089	0.0087
36	Undecane	C_11_H_24_	18.52	0.0067	0.0102	0.0098	ND	ND	ND	0.0051	0.0092	0.0087	0.0091	0.0134	0.0125
37	Tridecane	C_13_H_28_	26.76	0.0035	0.0048	0.0054	0.0036	0.0044	0.0047	0.0040	0.0063	0.0059	0.0053	0.0080	0.0084
38	3-Heptene, 2,2,4,6,6-pentamethyl-	C_12_H_24_	28.18	0.0023	0.0059	0.0071	0.0017	0.0040	0.0054	0.0074	0.0124	0.0148	0.0032	0.0123	0.0151
39	2-Methylundecane	C_12_H_26_	28.39	0.0269	0.0321	0.0346	0.0187	0.0219	0.0306	0.0317	0.0355	0.0572	0.0196	0.0241	0.0369
40	2-Pentene, 2,4,4-trimethyl	C_8_H_16_	29.22	0.0338	0.0322	0.0381	0.0114	0.0140	0.0178	0.0646	0.0748	0.0790	0.0106	0.0154	0.0166
41	Tetradecane	C_14_H_30_	31.12	0.0415	0.0358	0.0359	0.0393	0.0295	0.0385	0.0500	0.0682	0.0694	0.0448	0.0633	0.0568
42	2,4,6-Trimethyldecane	C_13_H_28_	31.21	0.0032	0.0031	0.0034	0.0023	0.0026	0.0031	0.0055	0.0058	0.0064	0.0028	0.0055	0.0058
43	Nonadecane	C_19_H_40_	34.9	0.0080	0.0082	0.0083	0.0074	0.0086	0.0091	0.0092	0.0105	0.0121	0.0097	0.0094	0.0104
44	Pentadecane	C_15_H_32_	35.02	0.0038	0.0051	0.0058	0.0021	0.0027	0.0035	0.0029	0.0052	0.0064	0.0017	0.0031	0.0035
45	Hexadecane	C_16_H_34_	39.02	0.0045	0.0041	0.0048	0.0045	0.0049	0.0047	0.0040	0.0049	0.0067	0.0139	0.0134	0.0133
46	Octadecane, 3-ethyl-5-(2-ethylbutyl)	C_26_H_54_	40.21	0.0020	0.0024	0.0023	0.0039	0.0033	0.0031	0.0048	0.0037	0.0036	0.0040	0.0026	0.0037
47	Octadecane	C_18_H_38_	41.1	0.0093	0.0137	0.0132	0.0083	0.0097	0.0149	0.0092	0.0191	0.0215	0.0096	0.0102	0.0098

* Relative peak area against an internal standard. ND—the compound was not detected.

## Data Availability

All datasets generated for this study are included in the manuscript/Supplementary Files.

## Supplementary Materials

The following supporting information can be downloaded at: https://www.mdpi.com/article/10.3390/foods12020379/s1, Table S1: Concentration of viable cells of *Lactobacillus delbrueckii* subsp. *bulgaricus* and *Streptococcus salivarius* subsp. *thermophilus* and their ratios in yogurts obtained with combination KM1 (log cfu/mL); Table S2: Titratable acidity and coagulation time of yogurts obtained with combination KM1; Table S3: Concentration of viable cells of *Lactobacillus delbrueckii* subsp. *bulgaricus* and *Streptococcus salivarius* subsp. *thermophilus* and their ratios in yogurts obtained with combination KM2 (log cfu/mL); Table S4: Titratable acidity and coagulation time of yogurts obtained with combination KM2; Table S5: Concentration of viable cells of *Lactobacillus delbrueckii* subsp. *bulgaricus* and *Streptococcus salivarius* subsp. *thermophilus* and their ratios in yogurts obtained with combination KM3 (log cfu/mL); Table S6: Titratable acidity and coagulation time of yogurts obtained with combination KM3; Table S7: Concentration of viable cells of *Lactobacillus delbrueckii* subsp. *bulgaricus* and *Streptococcus salivarius* subsp. *thermophilus* and their ratios in yogurts obtained with combination KM4 (log cfu/mL); Table S8: Titratable acidity and coagulation time of yogurts obtained with combination KM4; Table S9: Concentration of viable cells of *Lactobacillus delbrueckii* subsp. *bulgaricus* and *Streptococcus salivarius* subsp. *thermophilus* and their ratios in yogurts obtained with combination KM5 (log cfu/mL); Table S10: Titratable acidity and coagulation time of yogurts obtained with combination KM5; Table S11: Concentration of viable cells of *Lactobacillus delbrueckii* subsp. *bulgaricus* and *Streptococcus salivarius* subsp. *thermophilus* and their ratios in yogurts obtained with combination KM6 (log cfu/mL); Table S12: Titratable acidity and coagulation time of yogurts obtained with combination KM6.
