# Supplementary material for: Design and Volatile Compound Profiling of Starter Cultures for Yogurt Preparation
Source: Foods. 2023 Jan 13;12(2):379. doi: 10.3390/foods12020379 (PMC9858186; doi:10.3390/foods12020379)
Supplement: Supplementary file 1 [file foods-12-00379-s001.zip › foods-2125994-supplementary.pdf]

Table S1. Concentration of viable cells of *Lactobacillus delbrueckii* subsp. *bulgaricus* and *Streptococcus salivarius* subsp. *thermophilus* and their ratio in yogurts obtained with combination KM1 (log cfu/mL).

	**After inoculation**		**At coagulation**		**After 24 h**		
	L. bulgaricus	***S. thermophilus***	***L. bulgaricus***	***S. thermophilus***	***L. bulgaricus***	***S. thermophilus***	**Ratio rods : cocci**
Day 1	6.505	7.845	8.477	9.820	9.778	10.176	1:2.5
Day 2	7.799	7.869	9.176	9.301	9.857	10.301	1:2.7
Day 3	7.477	7.643	9.255	9.342	9.851	10.255	1:2.5
Day 4	7.447	7.708	9.477	9.613	9.863	10.301	1:2.7
Day 14	7.342	7.602	9.699	9.663	9.778	10.176	1:2.5
Day 15	7.732	7.799	9.415	9.708	9.839	10.279	1:2.8
Day 16	7.580	7.851	9.672	9.505	9.875	10.301	1:2.6
Day 17	7.833	7.919	9.322	9.613	9.681	10.079	1:2.5

Table S2. Titratable acidity and coagulation time of yogurts obtained with combination KM1.

	**After inoculation**	**After inoculation**	**After 24 h**	**Coagulation time**
Day 1	27 °T	61 °T	78 °T	3 h 10 min
Day 2	22 °T	70 °T	80 °T	3 h
Day 3	22 °T	70 °T	80 °T	3 h
Day 4	22 °T	70 °T	81 °T	3 h
Day 14	22 °T	70 °T	83 °T	3 h 15 min
Day 15	20 °T	68 °T	81 °T	3 h
Day 16	20 °T	70 °T	85 °T	3 h
Day 17	20 °T	69 °T	84 °T	3 h

Table S3. Concentration of viable cells of *Lactobacillus delbrueckii* subsp. *bulgaricus* and *Streptococcus salivarius* subsp. *thermophilus* and their ratio in yogurts obtained with a combination KM2 (log cfu/mL).

	**After inoculation**		**At coagulation**		**After 24 h**		
	L. bulgaricus	***S. thermophilus***	***L. bulgaricus***	***S. thermophilus***	***L. bulgaricus***	***S. thermophilus***	**Ratio rods : cocci**
Day 1	4.447	7.519	8.623	9.613	8.892	9.833	1:8.7
Day 2	4.301	7.146	8.602	9.301	8.857	9.813	1:9.0
Day 3	4.544	7.531	8.580	9.857	8.839	9.792	1:8.9
Day 4	5.892	7.740	8.602	9.544	8.845	9.806	1:9.1
Day 14	5.505	7.845	8.477	9.556	8.903	9.833	1:8.5
Day 15	5.799	7.869	8.398	9.447	8.857	9.792	1:8.6
Day 16	5.477	7.643	8.447	9.342	8.845	9.799	1:9.0
Day 17	5.447	7.708	8.477	9.613	8.875	9.820	1:8.8

Table S4. Titratable acidity and coagulation time of yogurts obtained with combination KM2.

	**After inoculation**	**After inoculation**	**After 24 h**	**Coagulation time**
Day 1	27 °T	67 °T	87 °T	2 h 45 min
Day 2	22 °T	68 °T	88 °T	2 h 45 min
Day 3	22 °T	66 °T	87 °T	2 h 40 min
Day 4	23 °T	67 °T	87 °T	2 h 40 min
Day 14	23 °T	66 °T	88 °T	3 h
Day 15	20 °T	67 °T	87 °T	2 h 40 min
Day 16	20 °T	68 °T	87 °T	2 h 45 min
Day 17	20 °T	66 °T	86 °T	2 h 40 min

Table S5. Concentration of viable cells of *Lactobacillus delbrueckii* subsp. *bulgaricus* and *Streptococcus salivarius* subsp. *thermophilus* and their ratio in yogurts obtained with combination KM3 (log cfu/mL).

	**After inoculation**		**At coagulation**		**After 24 h**		
	L. bulgaricus	***S. thermophilus***	***L. bulgaricus***	***S. thermophilus***	***L. bulgaricus***	***S. thermophilus***	**Ratio rods : cocci**
Day 1	5.820	7.602	7.301	9.556	7.477	9.653	1:150
Day 2	6.114	7.531	8.477	9.602	8.857	10.176	1:21
Day 3	5.778	7.322	8.079	9.699	8.362	9.914	1:36
Day 4	5.833	7.398	8.954	9.041	9.362	9.519	1:1.4
Day 14	5.342	6.845	8.477	10.415	9.778	10.716	1:8.6
Day 15	5.724	7.380	8.255	10.301	9.799	10.740	1:8.7
Day 16	6.301	7.643	8.763	10.342	9.778	10.716	1:8.6
Day 17	5.833	7.708	8.477	10.301	9.799	10.740	1:8.7

Table S6. Titratable acidity and coagulation time of yogurts obtained with combination KM3.

	After inoculation	After inoculation	After 24 h	Coagulation time
Day 1	27 °T	61 °T	68 °T	3 h 25 min
Day 2	21 °T	67 °T	76 °T	3 h 25 min
Day 3	22 °T	65 °T	75 °T	3 h 25 min
Day 4	23 °T	65 °T	74 °T	3 h 25 min
Day 14	22 °T	65 °T	83 °T	3 h
Day 15	20 °T	65 °T	82 °T	3 h
Day 16	20 °T	69 °T	84 °T	3 h
Day 17	20 °T	69 °T	82 °T	3 h

Table S7. Concentration of viable cells of *Lactobacillus delbrueckii* subsp. *bulgaricus* and *Streptococcus thermophilus* and their ratio in yogurts obtained with combination KM4 (log cfu/mL).

	After inoculation		At coagulation		After 24 h		
	L. bulgaricus	*S. thermophilus*	*L. bulgaricus*	*S. thermophilus*	*L. bulgaricus*	*S. thermophilus*	**Ratio rods : cocci**
Day 1	6.740	6.845	9.845	10.342	10.602	11.255	1:4.5
Day 2	7.633	7.477	9.813	10.875	10.623	11.301	1:4.7
Day 3	7.301	7.531	9.851	10.716	10.580	11.279	1:5.0
Day 4	7.447	7.613	9.919	10.613	10.623	11.322	1:5.0
Day 14	7.342	7.699	10.000	10.820	10.591	11.279	1:4.8
Day 15	7.633	8.380	10.176	10.519	10.681	11.380	1:5.0
Day 16	7.477	8.146	10.255	10.342	10.519	11.204	1:4.8
Day 17	7.462	8.491	10.114	10.663	10.633	11.322	1:5.0

Table S8. Titratable acidity and coagulation time of yogurts obtained with combination KM4.

	**After inoculation**	**After inoculation**	**After 24 h**	**Coagulation time**
Day 1	27 °T	61 °T	79 °T	3 h 10 min
Day 2	21 °T	65 °T	80 °T	3 h
Day 3	22 °T	64 °T	78 °T	3 h
Day 4	23 °T	64 °T	80 °T	3 h
Day 14	22 °T	65 °T	79 °T	3 h 10 min
Day 15	20 °T	64 °T	78 °T	3 h
Day 16	20 °T	66 °T	80 °T	3 h
Day 17	20 °T	66 °T	80 °T	3 h

Table S9. Concentration of viable cells of *Lactobacillus delbrueckii* subsp. *bulgaricus* and *Streptococcus salivarius* subsp. *thermophilus* and their ratio in yogurts obtained with combination KM5 (log cfu/mL).

	**After inoculation**		**At coagulation**		**After 24 h**		
	L. bulgaricus	***S. thermophilus***	***L. bulgaricus***	***S. thermophilus***	***L. bulgaricus***	***S. thermophilus***	**Ratio rods : cocci**
Day 1	4.491	6.301	6.301	9.204	6.778	9.653	1:750
Day 2	-	6.778	-	8.699	-	8.813	-
Day 3	-	6.146	-	8.623	-	8.778	-
Day 4	-	6.785	-	8.322	-	8.924	-
Day 14	-	6.491	-	9.322	-	9.833	-
Day 15	-	6.447	-	9.633	-	9.851	-
Day 16	-	6.748	-	9.785	-	9.919	-
Day 17	-	6.663	-	9.756	-	9.869	-

Table S10. Titratable acidity and coagulation time of yogurts obtained with combination KM5.

	After inoculation	After inoculation	After 24 h	Coagulation time
Day 1	27 °T	60 °T	67 °T	5 h 15 min
Day 2	20 °T	55 °T	62 °T	6 h 15 min
Day 3	20 °T	55 °T	63 °T	6 h 15 min
Day 4	20 °T	56 °T	64 °T	6 h 15 min
Day 14	21 °T	56 °T	64°T	6 h 10 min
Day 15	20 °T	54 °T	63 °T	6 h 15 min
Day 16	20 °T	53 °T	64 °T	6 h 15 min
Day 17	20 °T	55 °T	64 °T	6 h 15 min

Table S11. Concentration of viable cells of *Lactobacillus delbrueckii* subsp. *bulgaricus* and *Streptococcus salivarius* subsp. *thermophilus* and their ratio in yogurts obtained with combination KM6 (log cfu/mL).

	**After inoculation**		**At coagulation**		**After 24 h**		
	L. bulgaricus	***S. thermophilus***	***L. bulgaricus***	***S. thermophilus***	***L. bulgaricus***	***S. thermophilus***	**Ratio rods : cocci**
Day 1	5.716	6.623	7.079	9.204	7.477	10.301	1:667
Day 2	4.799	7.732	6.740	9.301	7.079	10.398	1:2038
Day 3	5.000	6.699	7.255	9.079	7.362	9.342	1:95.6
Day 4	4.892	7.041	7.322	8.322	7.633	8.908	1:18.8
Day 14	5.079	7.477	7.477	10.663	7.778	10.875	1:1250
Day 15	4.799	7.732	6.929	10.531	7.623	10.653	1:1071
Day 16	5.230	7.633	7.255	10.342	7.724	10.544	1:663
Day 17	5.380	7.613	7.477	10.623	7.633	10.785	1:1418

Table S12. Titratable acidity and coagulation time of yogurts obtained with combination KM6.

	**After inoculation**	**After inoculation**	**After 24 h**	**Coagulation time**
Day 1	27 °T	62 °T	68 °T	4 h
Day 2	20 °T	60 °T	70 °T	3 h 45 min
Day 3	21 °T	61 °T	70 °T	3 h 45 min
Day 4	21 °T	60 °T	71 °T	3 h 45 min
Day 14	21 °T	60 °T	74 °T	3 h 40 min.
Day 15	20 °T	61 °T	75 °T	3 h 45 min
Day 16	20 °T	62 °T	74 °T	3 h 40 min
Day 17	20 °T	62 °T	76 °T	3 h 45 min
